# Supplementary material for: A constricted mitochondrial morphology formed during respiration
Source: Nat Commun. 2025 Jul 1;16:5314. doi: 10.1038/s41467-025-60658-9 (PMC12215465; doi:10.1038/s41467-025-60658-9)
Supplement: Supplementary file 1 — Supplementary Information [file 41467_2025_60658_MOESM1_ESM.pdf]

Supplementary figure 1

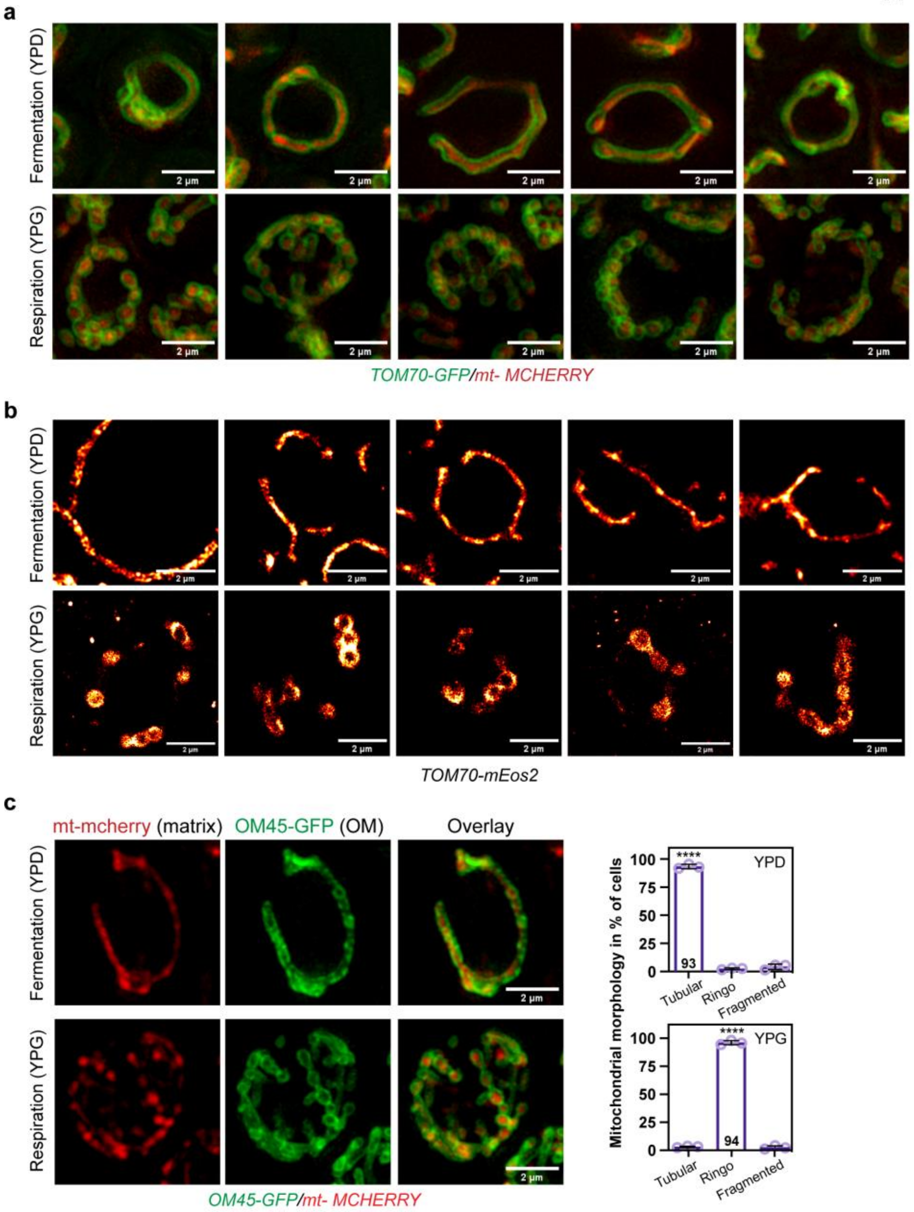

Supplementary Fig. 1: SIM and PALM detection of the Ringo mitochondrial morphology does not depend on the protein used to label OMs.

(a) Five distinct SIM acquisitions of cells (z-projections) labeled for mitochondrial matrix (mt-mCherry) and Outer Membranes (Tom70-GFP) in fermentation (top) or respiration (bottom). Scale bar, 2 µm. **(b)** Five distinct PALM images of cells labeled with TOM70-mEOS2 in fermentation (top) or respiration (bottom). Scale bar, 2 µm. **(c)** SIM acquisitions of cells labeled for mitochondrial matrix (mt-mCherry) and Outer Membranes (OM45-GFP) in fermentation (top) or respiration (bottom). Scale bar, 2 µm. Right graphs: percentage of cells with Tubular, Ringo or Fragmented mitochondria. Mean ± s.d. from >52 cells in n=3 independent experiments (purple circles). ****p <0.0001 (One-way Anova followed by Tukey's multiple comparisons test). Note that the respiratory Ringo morphology forms independent of the outer membrane protein used to label mitochondrial outer membranes (*i.e.* OM45 or Tom70).

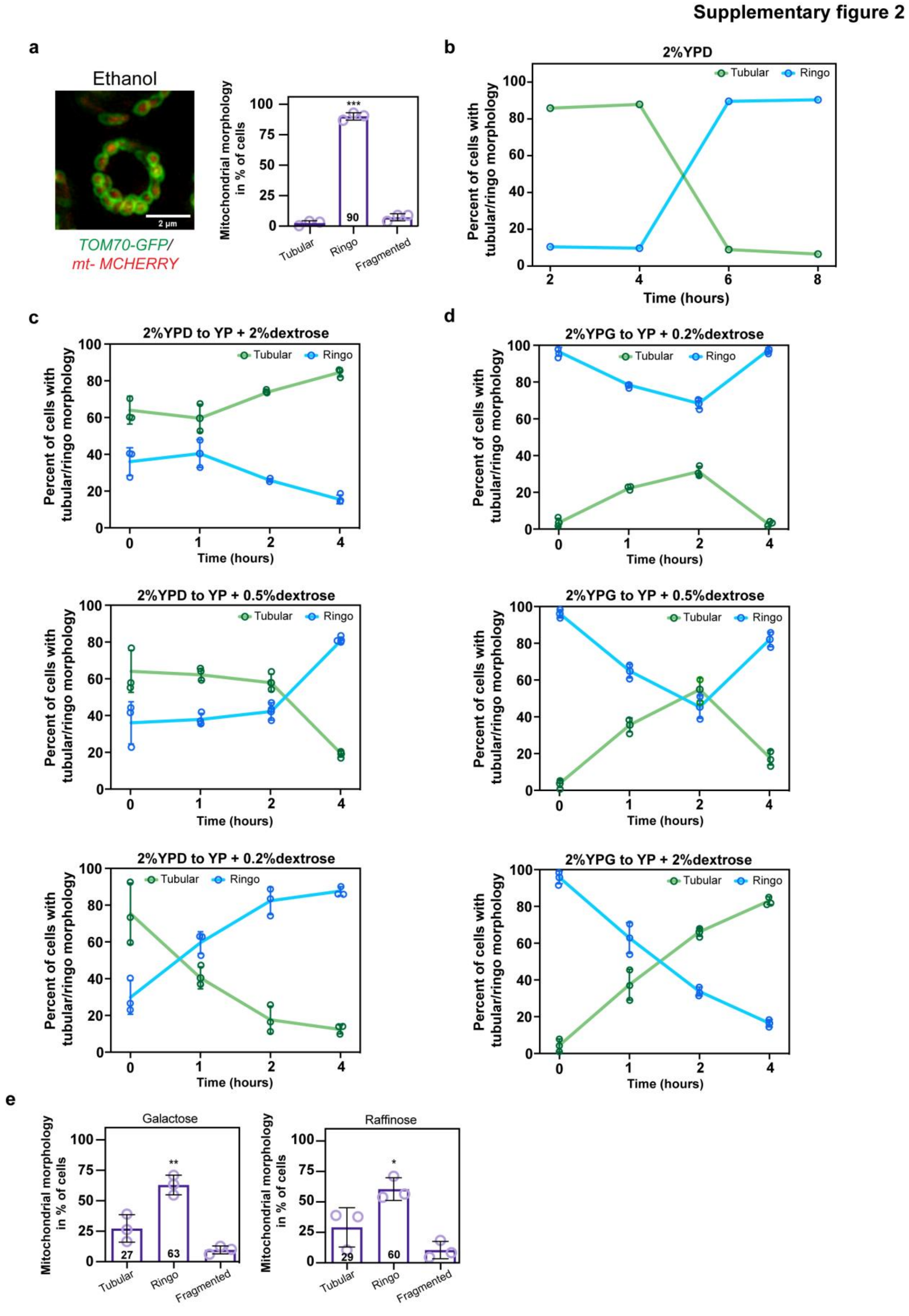
Supplementary figure 2
a
Ethanol
2 µm
TOM70-GFP/
mt- MCHERRY
Mitochondrial morphology in % of cells
***
90
Tubular
Ringo
Fragmented
b
2%YPD
Tubular
Ringo
Percent of cells with tubular/ringo morphology
Time (hours)
c
2%YPD to YP + 2%dextrose
2%YPD to YP + 0.5%dextrose
2%YPD to YP + 0.2%dextrose
d
2%YPG to YP + 0.2%dextrose
2%YPG to YP + 0.5%dextrose
2%YPG to YP + 2%dextrose
e
Galactose
**
27
63
Raffinose
*
29
60

Supplementary Fig. 2: The Ringo morphology dominates in all respiratory conditions.

(a) SIM acquisitions of *WT* cells labeled for mitochondrial matrix (mt-mCherry) and Outer Membranes (Tom70-GFP) in Ethanol media. Scale bar, 2 µm. Right graph: percentage of cells with Tubular, Ringo or Fragmented mitochondria. Mean ± s.d. from >193 cells in n=3 independent experiments (purple circles). ****p <0.0001 (One-way Anova followed by Tukey's multiple comparisons test). Note that the Ringo morphology dominates in the Ethanol respiratory media. **(b)** Percentage of WT cells labeled with mt-mCherry and Tom70-GFP with Tubular or Ringo mitochondria during 8 hours culture in 2% YPD. Mean from >78 cells per time point. Note that mitochondrial morphology progressively switches from Tubular to Ringo after 4 hours of culture which is presumably caused by progressive dextrose depletion. **(c)** Percentage over 4 hours of WT cells (labeled with mt-mCherry and Tom70-GFP) with Tubular or Ringo mitochondria. Cells were pre-cultured in 2% YPD (fermentative) and switched to YP media with decreasing dextrose concentrations (2%, 0.5%, and 0.2%). Mean ± s.d. from >44 cells per time point in n=3 independent experiments (colored circles). Note that the lower the glucose concentration, the faster the switch from Tubular to Ringo morphology occurs. **(d)** Same as (c) but with cells pre-cultured in 2% YPG (respiration) and switched to YP media containing increasing concentrations of glucose (0.2%, 0.5%, and 2%). Mean ± s.d. from >57 cells per time point in n=3 independent experiments (colored circles). Note that the higher the glucose concentration, the faster the switch from Ringo to Tubular morphology. **(e)** Percentage of WT cells (labeled with mt-mCherry and Tom70-GFP) with Tubular, Ringo or Fragmented mitochondria in Galactose (left) and Raffinose (right) media. Mean ± s.d. from >88 cells in n=3 independent experiments (purple circles). **p =0.0044, *p =0.036 * (One-way Anova followed by Tukey's multiple comparisons test).

Supplementary figure 3

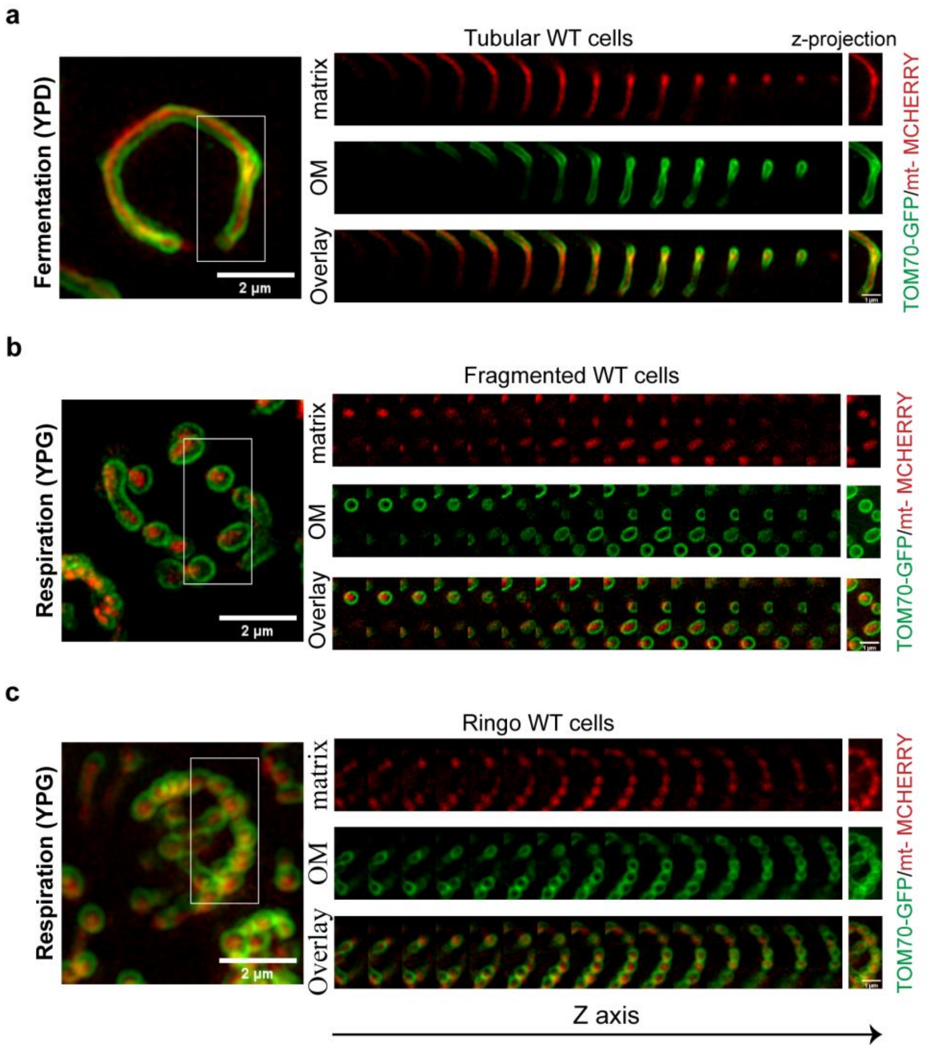

Supplementary Fig. 3: Matrix within Ringo mitochondria is continuous.

Maximum intensity projection (MIP) of z-stack SIM acquisitions from cells labeled for mitochondrial matrix (mt-mCherry) and Outer Membranes (Tom70-GFP) with distinct mitochondrial morphologies. Series represents top-to-bottom individual slices of the stack along Z-axis. MIP of inset is shown at the end of each row. Scale bar, 2 µm and for inset, 1 µm. **(a)** Tubular morphology during fermentation. The matrix in red in continuous. **(b)** Fragmented morphology during respiration. The matrix in red is not continuous. **(c)** Ringo morphology during respiration. The matrix in red seems continuous.

Supplementary figure 4

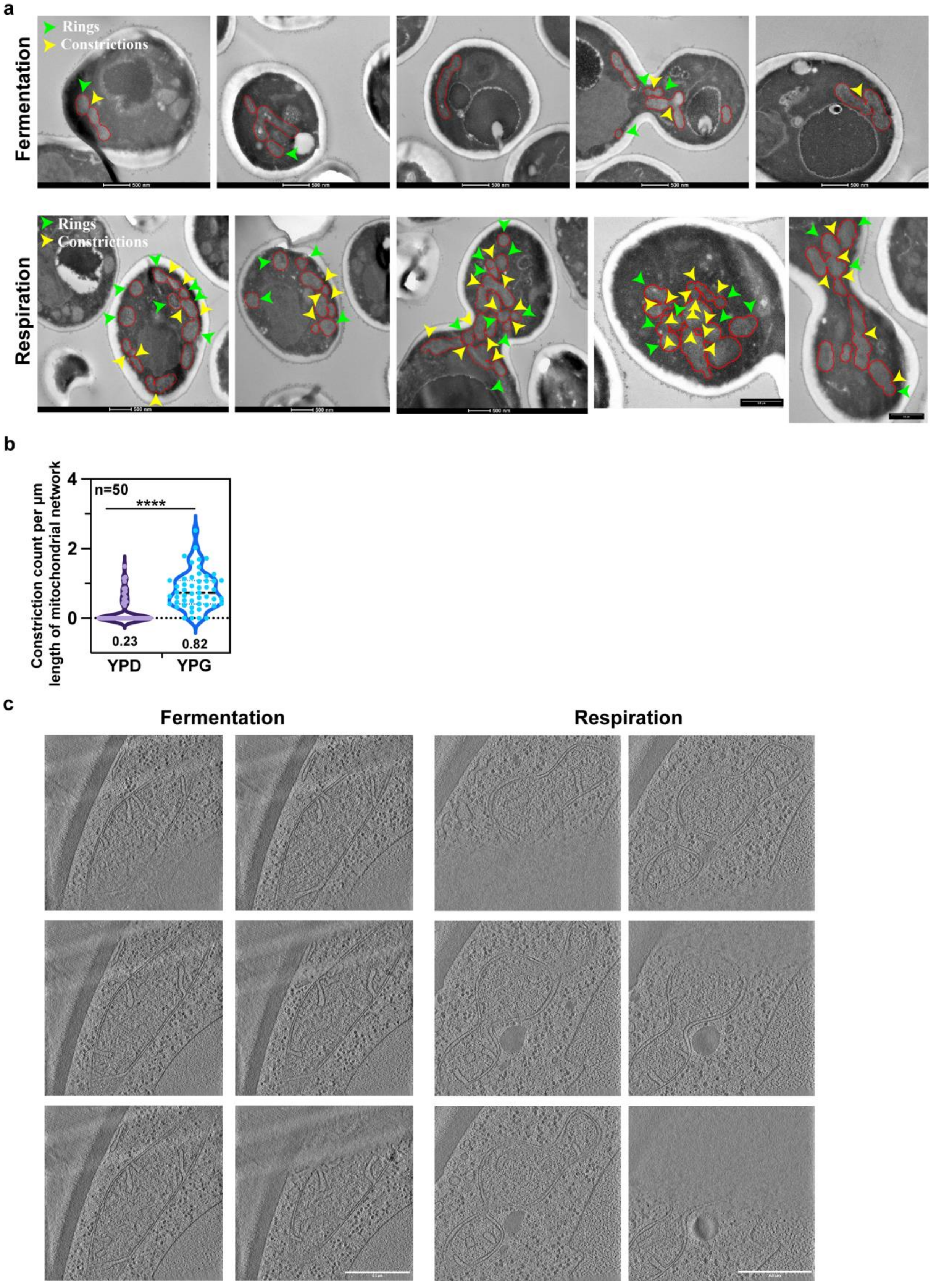

Supplementary Fig. 4: Transmission Electron Microscopy (TEM) and Cryo-Electron Tomography (Cryo-ET) reveal and confirm ultrastructural differences between Tubular and Ringo mitochondrial networks.

(a) Various examples of TEM micrographs of cells in fermentation (Scale bar, 1 µm) or respiration (Scale bar, 1 µm or 500 nm). Mitochondria are delimited by red demarcations. Rings and constrictions are indicated by green or yellow arrowheads, respectively. Note the strong increase of mitochondrial constrictions within Ringo mitochondrial networks. **(b)** Average constriction counts per µm length of mitochondrial network in YPD or YPG as quantified in TEM. Violin plots from n=50 cells with mean indicated at the bottom and by black dashed lines (quartiles with dotted lines). ****p <0.0001 (Two-tailed Mann whitney test) **(c)** Top to bottom slices through tomographic volumes of cell in fermentation (left) and respiration (right). Scale bars, 500 nm.

Supplementary figure 5

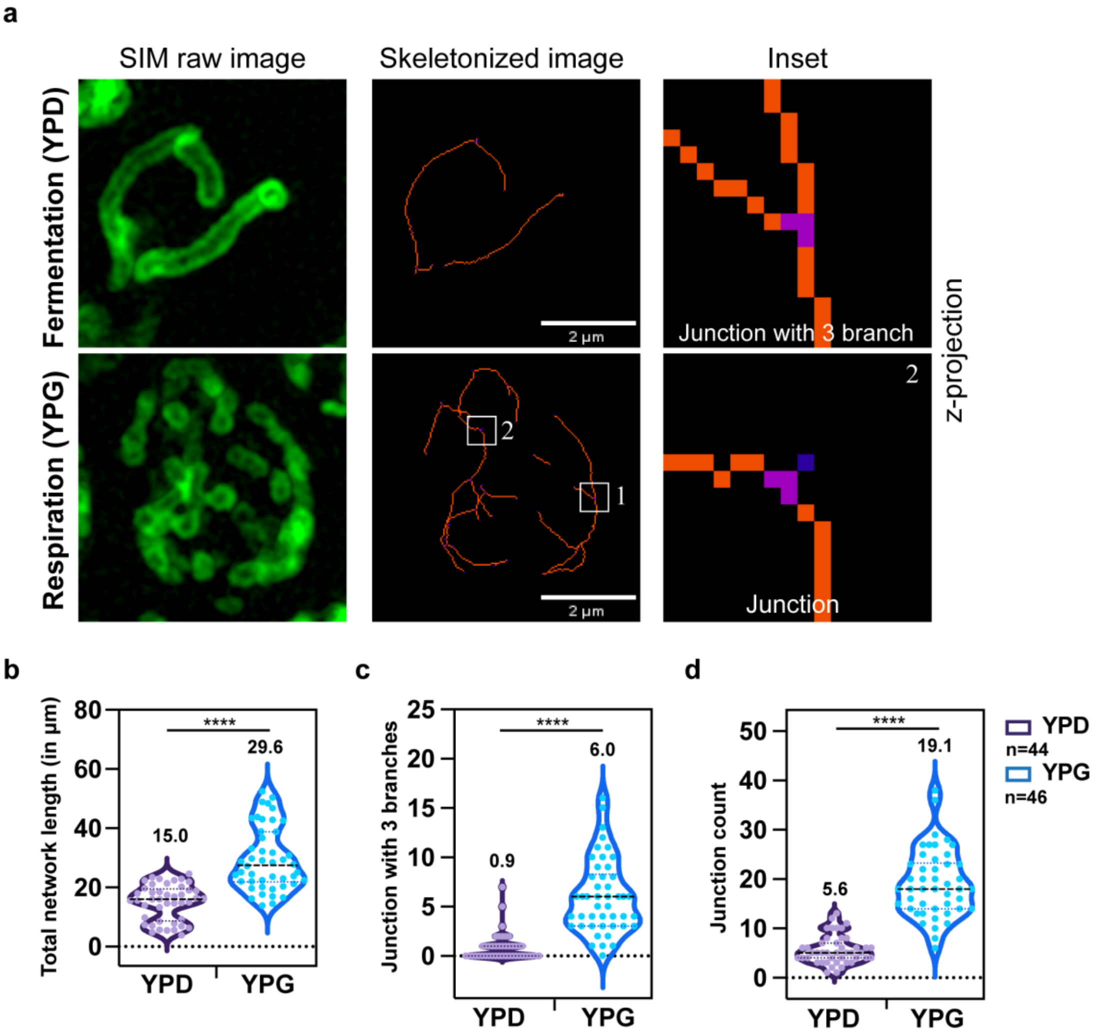

Supplementary Fig. 5: Mitochondrial networks are denser and more branched in respiration. (a) Column1: SIM Z-projection of *TOM70-GFP* cells in fermentation and respiration. Column 2: Skeletonized images from column 1 generated using analyzed skeleton plugin. Column 3: Expanded views of junction regions numbered in column 2. Scale bar, 2µm. (b) Total mitochondrial networks length, (c) junction count and (d) junction with 3 branches from skeletonized images in YPD and YPG. Violin plots from n=44 cells in YPD (purple) and n=46 cells in YPG (blue) with mean indicated at the top and by dashed lines (quartiles with dotted lines). ****p <0.0001 (Two-tailed unpaired T-test). Note that Ringo networks are denser and more branched than tubular networks which is consistent with previous observation in respiration against fermentation[1].

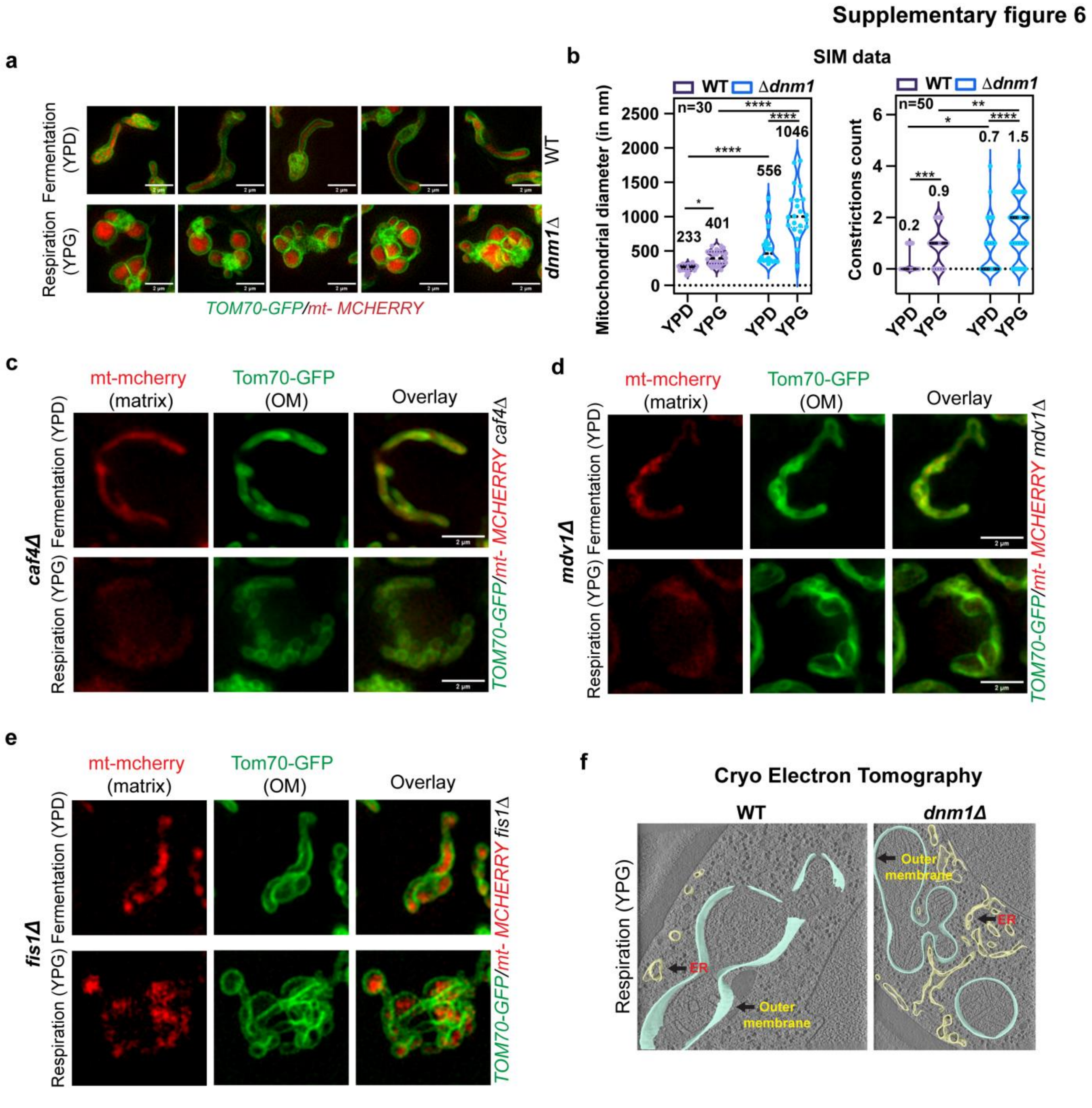

Supplementary Fig. 6: The HFR mitochondrial morphology is generated in the absence of either Dnm1, Mdv1 or Fis1.

(a) Five distinct SIM projections of *WT* or *dnm1Δ* cells labeled for mitochondrial matrix (mt-mCherry) and Outer Membranes (Tom70-GFP) in fermentation (top) or respiration (bottom). Scale bar, 2 µm. **(b)** Average mitochondrial diameter (left, n=30 cells), and constriction counts per cell (right, n=50 cells) as quantified in SIM acquisitions from *WT* (purple) or *dnm1Δ* (blue) cells in YPD or YPG. Violin plots with mean indicated at the top and by dashed lines (quartiles with dotted lines). ****p <0.0001, *p =0.0184, *p =0.0168, ***p =0.0007, **p =0.001 (Two-way Anova followed by Tukey's multiple comparisons test). Note that mitochondria with the

HFR morphology are distinct from mitochondria with Ringo morphology in terms and diameter and amount of apparent constrictions. **(c, d and e)** SIM projections of *caf4Δ* (b), *mdv1Δ* (c) or *fis1Δ* (d) cells labeled for mitochondrial matrix (mt-mCherry) and Outer Membranes (Tom70-GFP) in fermentation (top) or respiration (bottom). Scale bar, 2 µm. Note that the HFR morphology is seen in the absence of Mdv1 or Fis1 but not in the absence of Caf4 where Ringo networks dominate. **(f)** Slices through tomographic volumes and 3D renderings of Ringo or HFR mitochondria from *WT* and *dnm1Δ* cells, respectively. Endoplasmic Reticulum (ER) and mitochondrial outer membranes are indicated. Scale bars, 500 nm. The WT 3D rendering is the same as in Fig. 1e YPG but with ER indicated. The *dnm1Δ* 3D rendering is the same as in Fig. 2e YPG but with distinct colors and orientation. Note the presence of ER at HFR but not at Ringo constrictions. See also Fig. 2i and 2j.

Supplementary figure 7

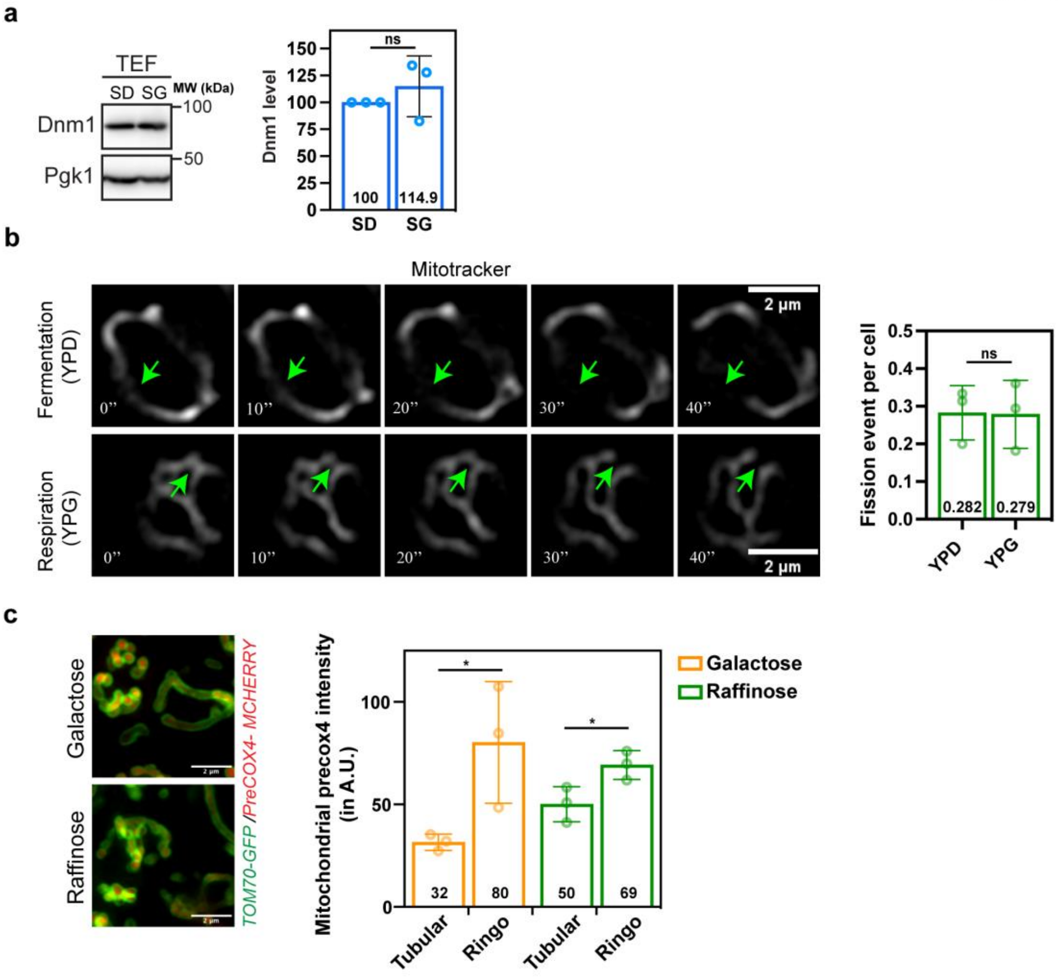

Supplementary Fig. 7: Mitochondrial fission efficiency in dextrose or glycerol media and mitochondrial import in galactose or raffinose media.

(a) Total protein extracts prepared from *dnm1Δ* cells transformed with the *TEF-DNM1* plasmid in SD or SG and analyzed by immunoblotting as indicated. MW (kDa) indicated on the right. Right graph: Dnm1 levels normalized to Pgk1 in SD or SG. Mean ± s.d. from n=3 independent experiments (blue circles). ns *p* =0,4127, not significant (Two-tailed unpaired t-test). Note that the level of Dnm1 expressed under control of the *TEF* promoter does not increase in respiratory as compared to fermentative growth. **(b)** Screenshot of SIM Time-lapse from Dnm1-mcherry, Mmm1-GFP, mitotracker (grey) triple-labeled cells during fermentation and respiration. Green arrows indicate fission events. Quantitation of fission events per cell as mean ± s.d. from >51 cells in n = 3 independent experiments (green circles). ns *p* =0,4127, not significant (Two-tailed unpaired t-test). Note that Fission efficiency is equivalent in fermentation and respiration. **(c)** SIM projections of *WT* cells labeled for the mitochondrial import marker precox4-mcherry and Outer Membranes (Tom70-GFP) in Galactose and Raffinose media. Scale bar, 3 µm. Right graph: Mitochondrial precox-4 mcherry intensity (in Absolute Units) within Tubular and Ringo mitochondria in galactose (yellow) and raffinose (green) media. Mean ± s.d. from >71 cells in n=3 independent experiments (colored circles). **p* =0.0481, **p* =0.0409 (Two-tailed unpaired t-test). Note that Ringo mitochondria that respire show increased transport of precox-4 mcherry as compared to tubular mitochondria that do not respire.

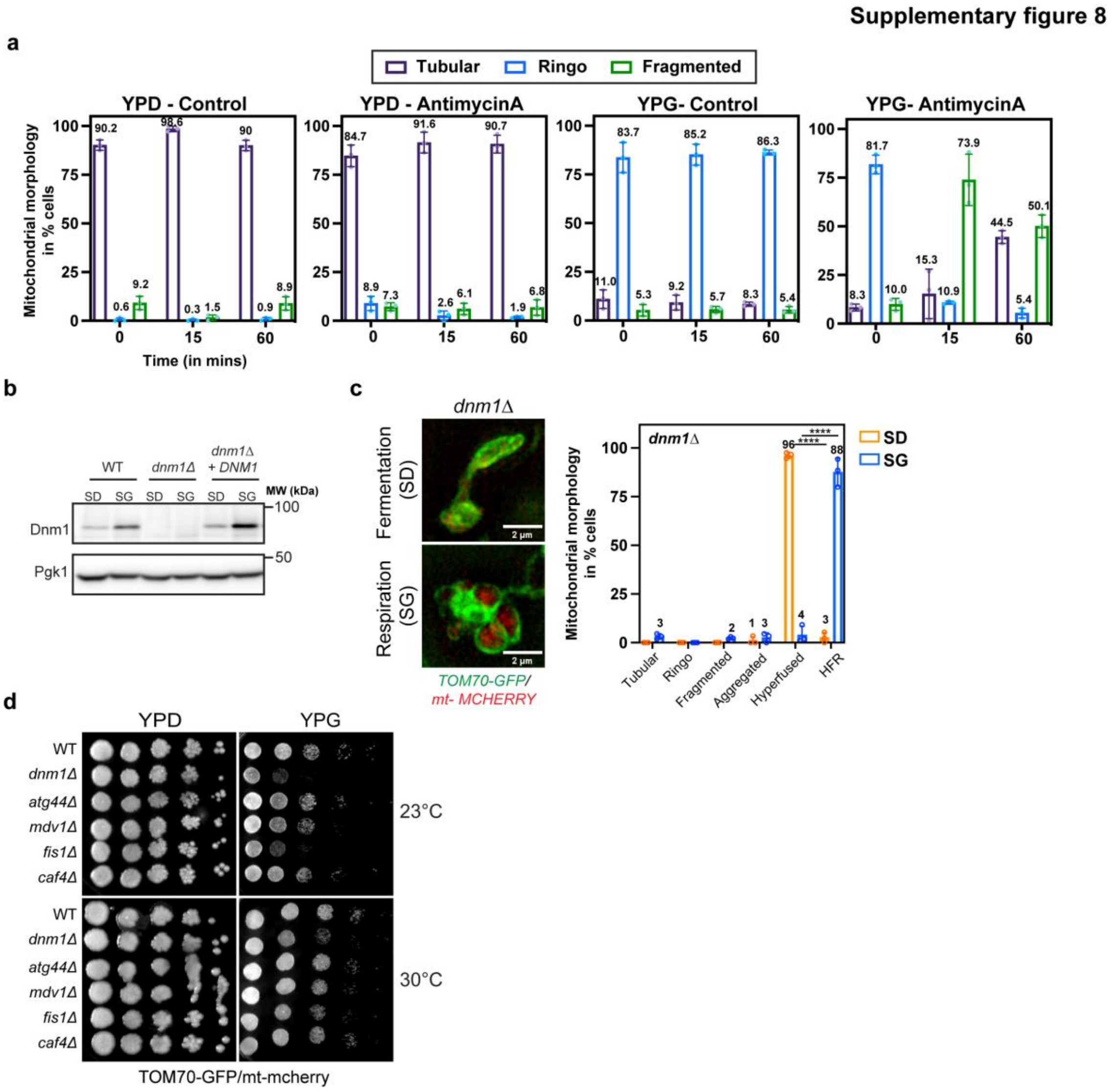

Supplementary Fig. 8: Related to Figure 4.

(a) Percentage of cells with Tubular (purple), Ringo (blue) or Fragmented (green) mitochondria in control or AntimycinA (1µM) treated cells during fermentation (YPD) and respiration (YPG). Mean ± s.d. from >38 cells per time point in n=3 independent experiments (colored circles). Note that AntimycinA treatment induces fragmentation of Ringo networks. **(b)** Total protein extracts prepared from *WT*, *dnm1Δ* or *dnm1Δ+DNM1* cells in Dextrose (SD) or Glycerol (SG) media and analyzed by immunoblotting with anti-Dnm1 and anti-Pgk1. MW (kDa) indicated on the right. **(c)** SIM projections of *dnm1Δ* cells labeled for mitochondrial matrix (mt-mCherry) and Outer Membranes (Tom70-GFP) in fermentation (SD) or respiration (SG). Scale bar, 2 µm. Right: Percentage of cells with Tubular, Ringo, Fragmented, Aggregated, Hyperfused or HFR mitochondria. Mean ± s.d. from >37 cells in n=3 independent experiments (colored circles). ****p <0.0001 (2way Anova followed by Tukey's multiple comparisons test). **(d)** Dextrose (YPD) and Glycerol (YPG) serial dilutions of *WT*, *dnm1Δ*, *atg44Δ*, *mdv1Δ*, *fis1Δ* and *caf4Δ* strains at 23 and 30°C (see also Fig. 4f).

Supplementary figure 9

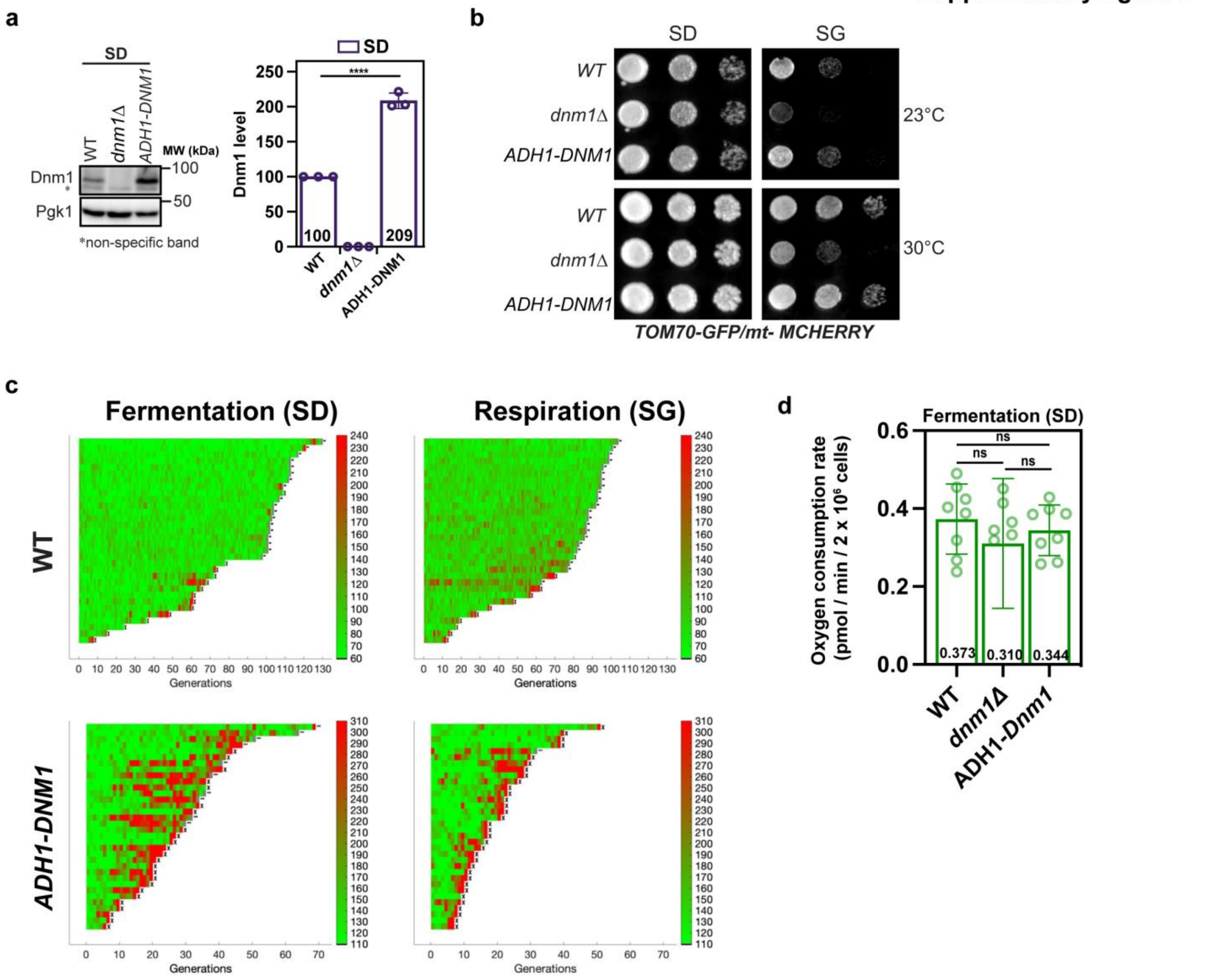

Supplementary Fig. 9: Related to Figure 5.

(a) Total protein extracts prepared from *WT, dnm1Δ* or *ADH1-DNM1* cells in dextrose (SD) media and analyzed by immunoblotting as indicated. MW (kDa) indicated on the right. Right graph: Dnm1 levels normalized to Pgk1 in *dnm1Δ* and *ADH1-DNM1* relative to the *WT* strains in SD. Mean ± s.d. from n=3 independent experiments (purple circles). ****p <0,0001 (One-way Anova followed by Tukey's multiple comparisons test) . Note the increase in ADH-driven expression of Dnm1 as compared to WT cells. **(b)** Dextrose (SD) and Glycerol (SG) serial dilutions of *WT*, *dnm1Δ* and *ADH1-DNM1* strains at 23 and 30°C. **(c)** *ADH-DNM1* cells exhibit a higher mortality rate as compared to *WT* cells exclusively in SG media. Microfludics results of independent lineages with the indicated genotypes. Cells were introduced into microcavities and cultured in SD or SG. Each horizontal line represents the consecutive cell cycles (generation) of a single lineage, and each segment corresponds to one cell cycle. An ellipsis (…) at the end of the lineage line indicates that the cell was living after the experiment, whereas an x indicates cell death. Cell cycle duration is indicated by the colored bar. (For SD media, *WT* n=32 cells and *ADH1-DNM1* n=32 cells; For SG media *WT* n=34 cells and *ADH1-DNM1* n=34 cells). **(d)**

Oxygen Consumption Rates (OCRs) of *WT*, *dnm1Δ* and *ADH-DNM1* cells grown in 2% Dextrose media (SD) at 30 °C. Mean ± s.d. from n=8 independent experiments (green circles). ns *p* =0,5392, ns *p* =0,8664, ns *p* =0,8313 ns, not significant (Two-tailed unpaired t-test) .

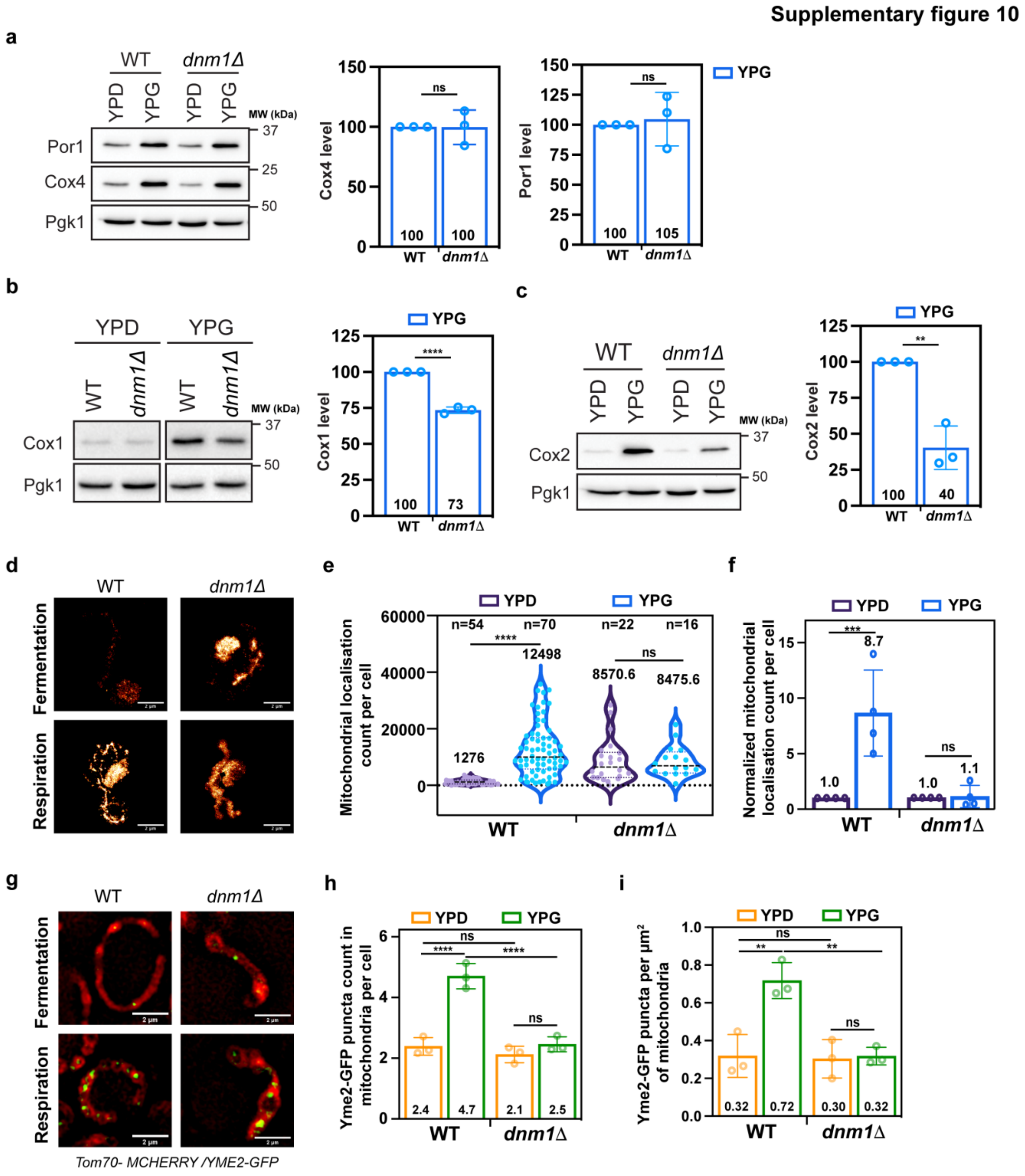

Supplementary Fig. 10: Related to Figure 6.

(a-c) Total protein extracts prepared from *WT* and *dnm1Δ* cells in YPD or YPG analyzed by immunoblotting as indicated. MW (kDa) on the right. Right graphs: Cox4 (a, left), Por1 (a, right), Cox1 (b) and Cox2 (c) levels normalized to Pgk1 in *dnm1Δ* relative to the *WT* strains in SG. Mean ± s.d. from n=3 independent experiments (blue circles). ns p =0.97 (a), ns p =0.7356 (a), ****p <0.0001 (b) **p =0,0024 (c), ns, not significant (Two-tailed unpaired t-test). **(d)**

Mitochondrial-DNA staining using Hoechst-PAINT in *WT* or *dnm1Δ* cells during fermentation and respiration. Scale bar, 2 µm. **(e)** Absolute count of Hoechst mitochondrial localizations per *WT* and *dnm1Δ* cells during fermentation (YPD) and respiration (YPG) from 4 independent experiments (Number n of cells analyzed are indicated). Violin plots with mean indicated at the top and by dashed lines (quartiles with dotted lines). ****p <0.0001, ns p >0.9999 **(f)** Same as (e) comparing the amounts of localizations in YPG relative to YPD. Mean ± s.d. from the 4 independent experiments (blue circles). **p =0.0008, ns p =0.9998. **(g)** SIM projections of *WT* and *dnm1Δ* cells labeled for the mtDNA marker Yme2 (*YME2-GFP*) and Outer Membranes (Tom70-mCherry) in fermentation (top) or respiration (bottom). Scale bar, 2 µm. ****p <0,0001, ns p =0.7382, ns p =0.5803 **(h)** Quantification of Yme2-GFP puncta count in Tom70-mCherry mitochondria per cell **(i)** Yme2-GFP puncta count per area of mitochondria. Mean ± s.d. from n=3 independent experiments (>50 cells per experiment). **p =0.0033, ns p =0.9968, ns p =0.9971. In (e-f) and (h-i), ns, not significant (2way Anova followed by Tukey's multiple comparisons test).

Supplementary Movie 1: 3D reconstruction of mitochondrial outer membranes from *WT* cells in fermentation or respiration. 3D stack of GFP channel acquired by SIM of TOM70-GFP wild-type cells grown in YPD (top row) and YPG (bottom). Cells grown in YPD (fermentation) show tubular phenotypes whereas cells grown on YPG media (respiration) display outer membrane reorganization resulting in the Ringo phenotype. Scale bar, 5 µm.

Supplementary Movie 2: 3D reconstruction of mitochondrial outer membranes from dnm1Δ cells in fermentation or respiration. 3D stack of GFP channel acquired by SIM of TOM70-GFP *dnm1Δ* cells grown in YPD (top row) and YPG (bottom). Cells grown in YPD (fermentation) show hyperfused phenotypes whereas cells grown on YPG media (respiration) display outer membrane reorganization resulting in the HFR phenotype. Scale bar, 5 µm.

Supplementary Table 1.

Strains Used in the Study

**Background and number**	Yeast Name	**Genotype**	**Occurrence in the study**	**Reference**
W303 (MCY553)	WT	Mat a, *ura3-1 trp1-1 leu2-3,112 his3-11,15 can1-100 RAD5 ADE2*	1d-h, 2f-j, S4a-c, S6f	
W303 (MCY1949)	*TOM70-GFP/mt- MCHERRY*	Mat a, *TOM70-GFP::CaURA3 ura3-1 trp1-1, mt-mcherry::LEU2,112 his3-11,15 can1-100 RAD5 ADE2*	1a-b, 2a, 4c-f, 5a-e, 6a-d, S1a, S2, S3, S5, S6a-b, S7c, S8a-b, S S9, S10a-f,	This study
W303 (MCY1497)	*TOM70-mEos2*	Mat a, *ura3-1 trp1-1 leu2-3,112 his3-11,15 can1-100 RAD5 ADE2 TOM70::mEos2-KanMX6*	1c, S1b	This study
W303 (MCY1940)	*OM45-GFP/mt- MCHERRY*	Mat a, *OM45-GFP::KanMX6 ura3-1 trp1-1 mt-MCHERRY ::LEU2,112 his3-11,15 can1-100 RAD5 ADE2*	S1c	This study
W303 (MCY1940)	*TOM70-GFP /mt- MCHERRY/ dnm1Δ*	Mat a, *TOM70-GFP::CaURA3 ura3-1 DNM1::TRP1, mt- MCHERRY ::LEU2,112 his3-11,15 can1-100 RAD5 ADE2*	2a, 2e-j, 3b, 4c-d, 4f, 5a, 5c, 5e, S6a-b, S6f, S8c, S8d, S9a-b, S9d, S10a-f	This study
W303 (MCY2099)	*TOM70-GFP/mt- MCHERRY caf4Δ*	Mat a, *CAF4::NatMX4 TOM70-GFP::CaURA3 ura3-1 trp1-1, mt- MCHERRY ::LEU2,112 his3-11,15 can1-100 RAD5 ADE2*	2b, 4f, S6c, S8d	This study
W303 (MCY2097)	*TOM70-GFP::mt- MCHERRY mdv1Δ*	Mat a, *MDV1::NatMX4 TOM70-GFP::CaURA3 ura3-1 trp1-1, mt- MCHERRY ::LEU2,112 his3-11,15 can1-100 RAD5 ADE2*	2c, 4f, S6d, S8d	This study
W303 (MCY 2204)	*TOM70-GFP::mt- MCHERRY fis1Δ*	Mat a, *FIS1::NatMX4 TOM70-GFP::CaURA3 ura3-1 trp1-1, mt- MCHERRY ::LEU2,112 his3-11,15 can1-100 RAD5 ADE2*	2d, 4f, S6e, S8d	This study
W303 (MCY 2373)	*TOM70-GFP::mt- MCHERRY atg44Δ*	Mat a, *ATG44::NatMX4 TOM70-GFP::CaURA3 ura3-1 trp1-1, mt- MCHERRY ::LEU2,112 his3-11,15 can1-100 RAD5 ADE2*	4c-f, S8d	This study
W303 (NBT1343)	*TOM70- MCHERRY /DNM1-GFP*	*TOM70:: MCHERRY -Nat DNM1::GFP-TRP ura3-1 trp1-1 leu2-3,1 12 his3-11,15 can1-100 RAD5 ADE2*	3a, 3c, 3f	This study
W303 (NBT344)	*TOM70- MCHERRY/MMM1-GFP*	*TOM70:: MCHERRY -Nat MMM1::GFP-TRP ura3-1 trp1-1 leu2-3,112 his3-11,15 can1-100 RAD5 ADE2*	3a, 3b, 3c, 3f	This study
W303 (MCY2199)	*Dnm1- MCHERRY /MMM1-GFP*	Mat a, *ura3-1 trp1-1 leu2-3,112 his3-11,15 can1-100 RAD5 ADE2; MMM1-GFP::TRP1 DNM1-MCHERRY:: NATNT2*	3d-e, S7b	This study
W303 (MCY1607)	*TOM70-GFP /PreCOX4-MCHERRY*	Mat a, *TOM70-GFP::CaURA3 PreCox4- MCHERRY ::HphMX ura3-1 trp1-1 leu2-3,112 his3-11,15 can1-100 RAD5 ADE2*	S7c	Gift from Zhou XU
W303 (MCY2166)	*TOM70-GFP/mt- MCHERRY+ pRS413*	Mat a, *ura3-1; trp1-1; leu2-3,112; his3-11,15; can1-100; RAD5; ADE2; MITO-MCHERRY::LEU2; TOM70-GFP::CaURA3; pRS413*	4a, S8b	This study

W303 (MCY2169)	*TOM70-GFP/mt- MCHERRY/ dnm1Δ + TEF-DNM1*	Mat a, *ura3-1; trp1-1; leu2-3,112; his3-11,15; can1-100; RAD5; ADE2; MITO-MCHERRY::LEU2; TOM70-GFP::CaURA3; DNM1::TRP1; p413TEF-DNM1*	S7a	This study
W303 (MCY2170)	*TOM70-GFP/mt- MCHERRY/ dnm1Δ +DNM1*	Mat a, *ura3-1; trp1-1; leu2-3,112; his3-11,15; can1-100; RAD5; ADE2; MITO-MCHERRY::LEU2; TOM70-GFP::CaURA3; DNM1::TRP1; pRS413-DNM1-DNM1*	4a-b, S8b	This study
W303 (MCY2171)	*TOM70-GFP/mt- MCHERRY/ dnm1Δ + pRS413*	Mat a, *ura3-1; trp1-1; leu2-3,112; his3-11,15; can1-100; RAD5; ADE2; MITO-MCHERRY::LEU2; TOM70-GFP::CaURA3; DNM1::TRP1; pRS413*	4a-b, S8b	This study
W303 (MCY 2290)	*ADH1-DNM1/ TOM70-GFP/mt- MCHERRY/*	Mat a, *ura3-1; trp1-1; leu2-3,112; his3-11,15; can1-100; RAD5; ADE2; MITO-MCHERRY::LEU2; TOM70-GFP::CaURA3; NatMX4-Prom ADH1::Prom DNM1*	5a-e, 6a-d, S9	This study
W303 (MCY 1984)	*YME2-GFP/TOM70-MCHERRY*	Mat a, *ura3-1; trp1-1; leu2-3,112; his3-11,15; can1-100; RAD5; ADE2; TOM70-MCHERRY:: NatMX4; YME2-GFP(G65T)::kanMX6*	S10g-i	This study
W303 (MCY 2018)	*YME2-GFP/TOM70-MCHERRY/ dnm1Δ*	Mat a, *ura3-1; trp1-1; leu2-3,112; his3-11,15; can1-100; RAD5; ADE2; TOM70-MCHERRY:: NatMX4; YME2-GFP(G65T)::kanMX6; DNM1::TRP1*	S10g-i	This study
W303 (MCY 2286)	*mtLACO-LACI-GFP*	Mat a, leu2-3,112; can1-100; ura3-1; ade2-1; his3-11,15; HO-P Cup-mt-3xGFP-LacI-HO	6e-g	Gift from Christof Osman
W303 (MCY 2297)	*ADH1-DNM1/mtLACO-LACI-GFP*	Mat a, leu2-3,112; can1-100; ura3-1; ade2-1; his3-11,15; HO-P Cup-mt-3xGFP-LacI-HO; *NatMX4-Prom ADH1::Prom DNM1*	6e-g	This study
W303 (MCY 2292)	*dnm1Δ/mtLACO-LACI-GFP*	Mat a, leu2-3,112; can1-100; ura3-1; ade2-1; his3-11,15; HO-P Cup-mt-3xGFP-LacI-HO; *DNM1:: HIS3MX6*	6e-g	This study

Supplementary Table 2.
Plasmids Used in the Study

**Name (Collection number)**	**Description**	**Reference**
pRS413	CEN, HIS3, Amp	2
p413TEF-*DNM1*	CEN, TEF promoter-DNM1, HIS3, Amp	This study
pRS413-*DNM1-DNM1*	CEN, DNM1 promoter-DNM1, HIS3, Amp	This study

1. Egner, A., Jakobs, S. & Hell, S. W. Fast 100-nm resolution three-dimensional microscope reveals structural plasticity of mitochondria in live yeast. *Proc Natl Acad Sci U S A* **99**, 3370–3375 (2002).
2. Sikorski, R. S. & Hieter, P. A system of shuttle vectors and yeast host strains designed for efficient manipulation of DNA in Saccharomyces cerevisiae. *Genetics* **122**, 19–27 (1989).
