# Supplementary material for: A constricted mitochondrial morphology formed during respiration
Source: Nat Commun. 2025 Jul 1;16:5314. doi: 10.1038/s41467-025-60658-9 (PMC12215465; doi:10.1038/s41467-025-60658-9)
Supplement: Supplementary file 3 — Description of Additional Supplementary Files [file 41467_2025_60658_MOESM3_ESM.pdf]

Description of Additional Supplementary Files

File Name: Supplementary Movie 1
Description: 3D reconstruction of mitochondrial outer membranes from WT cells in fermentation or respiration. 3D stack of GFP channel acquired by SIM of TOM70-GFP wild-type cells grown in YPD (top row) and YPG (bottom). Cells grown in YPD (fermentation) show tubular phenotypes whereas cells grown on YPG media (respiration) display outer membrane reorganization resulting in the Ringo phenotype. Scale bar, 5 µm.

File Name: Supplementary Movie 2
Description: 3D reconstruction of mitochondrial outer membranes from dnm1Δ cells in fermentation or respiration. 3D stack of GFP channel acquired by SIM of TOM70-GFP dnm1Δ cells grown in YPD (top row) and YPG (bottom). Cells grown in YPD (fermentation) show hyperfused phenotypes whereas cells grown on YPG media (respiration) display outer membrane reorganization resulting in the HFR phenotype. Scale bar, 5 µm.
